# Formation Mechanism of Ion Channel in Channelrhodopsin-2: Molecular Dynamics Simulation and Steering Molecular Dynamics Simulations

**DOI:** 10.3390/ijms20153780

**Published:** 2019-08-02

**Authors:** Ting Yang, Wenying Zhang, Jie Cheng, Yanhong Nie, Qi Xin, Shuai Yuan, Yusheng Dou

**Affiliations:** 1 Chongqing Key Laboratory of Big Data for Bio Intelligence, Chongqing University of Posts and Telecommunications, Chongqing 40065, China; 2 Department of Chemistry and Physical Sciences, Nicholls State University, P.O. Box 2022, Thibodaux, LA 70310, USA

**Keywords:** channelrhodopsin-2, photoisomerization, ion channel, hydrogen bond network, Steering Molecular Dynamics Simulations

## Abstract

Channelrhodopsin-2 (ChR2) is a light-activated and non-selective cationic channel protein that can be easily expressed in specific neurons to control neuronal activity by light. Although ChR2 has been extensively used as an optogenetic tool in neuroscience research, the molecular mechanism of cation channel formation following retinal photoisomerization in ChR2 is not well understood. In this paper, studies of the closed and opened state ChR2 structures are presented. The formation of the cationic channel is elucidated in atomic detail using molecular dynamics simulations on the all-trans-retinal (ChR2-trans) configuration of ChR2 and its isomerization products, 13-cis-retinal (ChR2-cis) configuration, respectively. Photoisomerization of the retinal-chromophore causes the destruction of interactions among the crucial residues (e.g., E90, E82, N258, and R268) around the channel and the extended H-bond network mediated by numerous water molecules, which opens the pore. Steering molecular dynamics (SMD) simulations show that the electrostatic interactions at the binding sites in intracellular gate (ICG) and central gate (CG) can influence the transmembrane transport of Na^+^ in ChR2-cis obviously. Potential of mean force (PMF) constructed by SMD and umbrella sampling also found the existing energy wells at these two binding sites during the transportation of Na^+^. These wells partly hinder the penetration of Na^+^ into cytoplasm through the ion channel. This investigation provides a theoretical insight on the formation mechanism of ion channels and the mechanism of ion permeation.

## 1. Introduction

It was found that channelrhodopsins (ChRs) could be expressed in neurons of living animals to mediate accurate and reliable control of action potential triggering in response to light pulses, without the need for exogenous retinal in vertebrate systems [1,2,3,4,5,6]. Deisseroth in 2005 used bacteriorhodopsin (BR) as a light-operated switch for brain cells to investigate the stimulation of neurons by photocurrent and proposed optogenetic techniques [1,2]. Since then, optogenetics technology has developed rapidly and been involved in many aspects of neuroscience research [7,8,9,10,11,12], including neural circuits, learning and memory, addiction, dyskinesias, sleep disorders, Parkinson’s disease, and animal models of depression and anxiety. Optogenetics is even expected to be used in the treatment of some diseases [13,14], such as severe epilepsy. ChRs are considered to be one of the most prominent optogenetic tools.

ChRs were initially described as an optically gated proton channel, but were later discovered to be capable of transporting univalent and divalent cations [15,16], with the following relative conductivities: H^+^ > Na^+^ > K^+^ > Ca^2+^ > Mg^2+^ [17,18,19,20,21]. A brief pulse of light triggers an influx of protons and sodium ions which lead to a photocurrent entering the cell [22]. Many efforts have been made to modify ChRs to be more light-sensitive and more selective to sodium than to protons [23]. One of the early modifications of ChRs involved replacement of histidine 134 with arginine, which reduces the inactivation of photocurrent and leads to higher ion selectivity and lower proton conductivity [3,24]. In 2014, as another regulation of neuronal cells, the negatively charged glutamate 90 was replaced with a positively charged arginine or lysine into the center of the channel to change ChRs from cationic to anionic conduction [25,26], thus inhibiting action potential. These application-specific variants will further contribute to the understanding of neurobiological circuits, but this much work remains to be done due to the lack of knowledge of the precise geometry of the selective filter.

**Figure 1:**
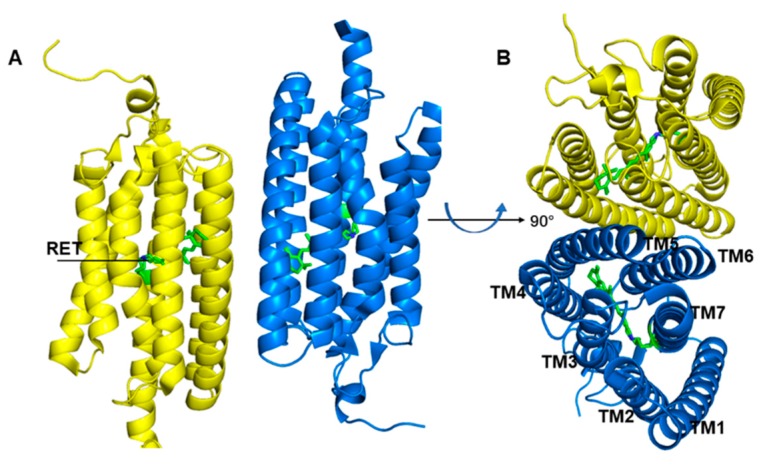
Overall structure presentation of the channelrhodopsin-2 (ChR2) dimer. (PDB ID: 6EID). (**A**) ChR2 side view. (**B**) ChR2 top view. The yellow and blue helixes represent different monomer respectively.

**Figure 2:**
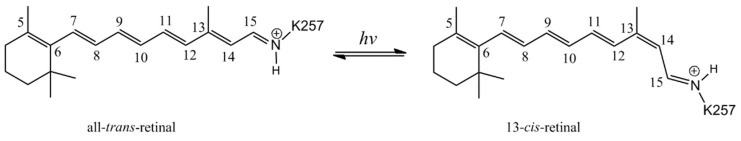
All-trans to 13-cis isomerization of the retinal chromophore in ChR2.

Channelrhodopsin-2 (ChR2), a representative protein of optogenetics technology, is a non-selective cationic channel protein that consists of seven transmembrane helices (TM1–TM7) (Figure 1), and the ion channel is located between TM1, TM2, TM3, and TM7. In the center of the TM domain of ChR2, a chromophore molecule, all-trans retinal (ATR), is covalently bound to K257 at TM7 and a protonated Schiff base is generated. The E123 and D253 residues play the roles of counterions to stabilize the positive charge of the Schiff base. Absorption of blue light converts ATR to the 13-cis configuration (Figure 2), and then triggers the photocycle reaction [17,27,28,29], which leads to a series of photochemical reactions and conformational transformations. During the photocycle, several intermediate states such as P500, P390, P520, and P480 are produced along with retinal isomerization [30,31,32]. P520 is a conductive state, which allows cations to flow into the cytoplasm from the extracellular matrix, and thus controls neuron activity. Therefore, ChR2 works as a light-gated cation channel and is widely used in light-induced neuronal depolarization and action potential studies.

The crystal structure of the ChR (a C1C2 chimera between ChR1 and ChR2 from *Chlamydomonas reinhardtii*) in a closed state was reported in 2012 [33]. Subsequent experimental [34] and theoretical studies [28,29] elucidated the formation of the C1C2 chimeric channel and revealed that cations can be transported through the cell membrane. However, the crystal structure of the ChR2 reported in 2018 [35] indicates considerable differences to C1C2 chimera: (1) the ion channel between TM1, TM2, TM3, and TM7 in ChR2 is respectively divided into four cavities by extracellular gate (ECG), central gate (CG), and intracellular gate (ICG). In the pores of the channel, R120, E90, and R268 are the cores of ECG, CG, and ICG respectively. In addition, there are a lot of water molecules in the channel. These key residues are bridge-linked by hydrogen bonding from water molecules, forming a complex hydrogen bonding network to block the ion channels. The three gates integrate into the complex hydrogen bond networks and control the closing and opening of the ion channel. On the other hand, only CG and ICG exist in C1C2. (2) the “DC gate” named based on D156 in TM4 and C128 in TM3, is located near the retinal molecule. There is a water-mediated hydrogen bond between D156 and C128 in ChR2. Water molecules far from the channel may spontaneously flow into the channel from solution to bridge an H-bond interaction between the residues D156 and C128. Moreover, it has a strong effect on the open channel lifetime even if the DC gates are not in the assumed ion pores. However, the hydrogen bond interaction between the D156 and C128 was not detected in the C1C2.

To understand these differences, the mechanism of the formation of cation channel in the ChR2 needs further investigation. Although the high-resolution ChR2 structure provides insights on the structures and functions of ChR2, the orientations of amino acids near retinal molecules after photoisomerization and its effect on channel function are still poorly understood. We conducted molecular dynamics simulations for ChR2-trans and ChR2-cis to clarify the mechanism of ion channel formation, so as to provide a theoretical basis for the design of novel light-controlled ion channel proteins.

## 2. Results and Discussion

### Formation of the Ion-Conducting Pore

ChR2 is a light-gated cation channel, where light excitation causes all-trans to 13-cis isomerization of retinal chromophore. This local conformational change triggers a cascade of events and leads to the preopen channel, the conducting state, desensitized structures, and, ultimately, back to the dark-adapted state [28]. It is suggested [24,28,29,34,36,37,38,39,40] that the residues E82, E83, E90, and E101 on TM2 are all involved in ion conduction of the channel. Among these, E90 is a key amino acid in the center of ion-conducting pores and plays an important role in the function of cation channels. Molecular dynamics simulations performed on ChR2-trans and ChR2-cis systems provides detailed information for further understanding the role of amino acids in channel function and the underlying mechanism of ion channel formation.

**Figure 3:**
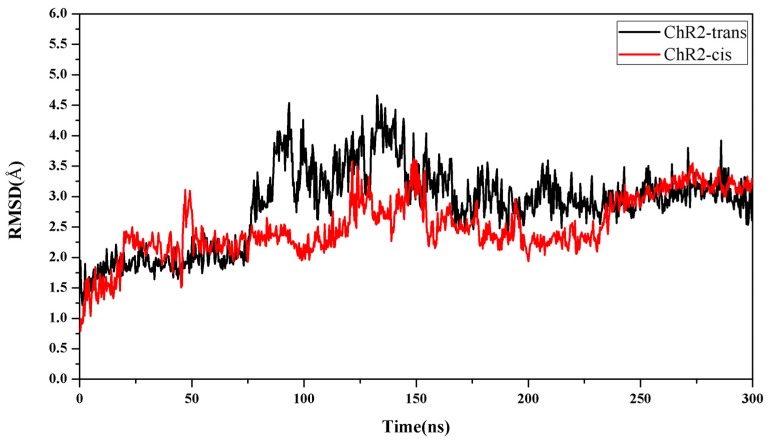
Time evolutions of root mean square difference (RMSD) of backbone atoms of ChR2-trans (black) and ChR2-cis (red).

The closed-state model was based on the ChR2 crystal structure. After the thermal equilibration, an unbiased MD production run of ChR2 was extended for 300 ns in the NPT ensemble. The protein structure tends to be stable, and the root mean square difference (RMSD) of the backbone atoms of the protein tends to fluctuate around 3 Å (Figure 3), indicating that the system has converged. The analysis of the closed-state structures was based on the trajectory of protein after equilibrium.

For the light-induced open state, simulation showed that the protein structure is essentially stable after 250 ns, and the RMSD is stable at a value of about 3.25 Å (Figure 3).

**Figure 4:**
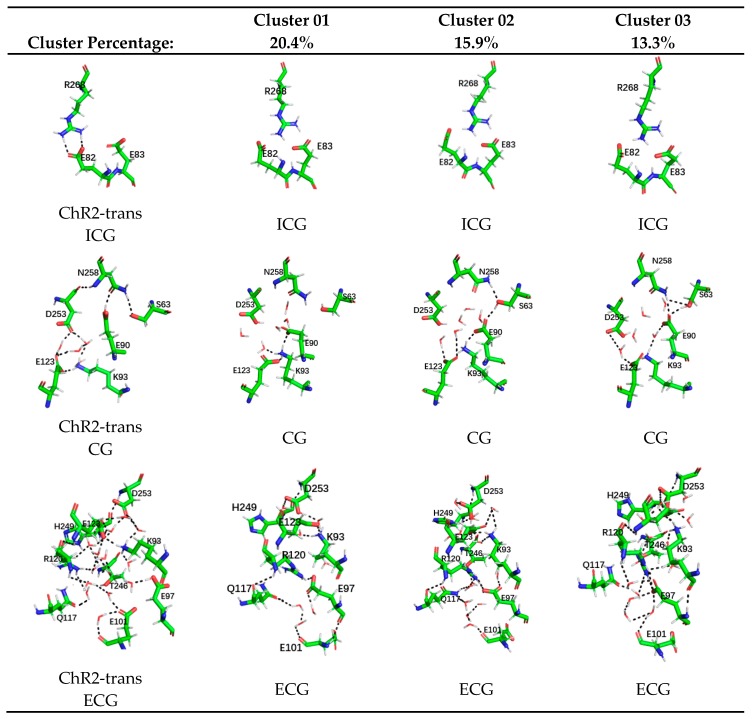
Cluster analysis of ChR2-cis systems. The first column shows the stable structures of the intracellular gate (ICG), central gate (CG), and extracellular gate (ECG), in the equilibrium trajectory of ChR2-trans. The second, third, and fourth columns are the representative conformations of the top three clusters in the ICG, CG, and ECG of ChR2-cis. The cluster percentages are also shown in the figure.

To explicitly show the differences in the conformations of different systems, the K-means algorithm in the MMTSB toolset [41] was used for the cluster analysis of resulted trajectories. The similar conformations were in the same group and their presentative conformations were picked up, as shown in Figure 4. In this figure the stable conformations of the ICG, CG, and ECG, in ChR2-trans are listed separately and the representative conformations of the ICG, CG, and ECG of only the top three clusters of ChR2-cis are shown.

In ICG, residues R268 in the structure represented by the top three cluster conformations of ChR2-cis are all shifted, resulting in the destruction of the hydrogen bonding originally formed with E82. For CG, the most obvious change is the first cluster conformation in ChR2-cis compared to that in ChR2-trans. This conformation shows that the hydrogen bonding interaction with N258 disappears and E90 flips down. The hydrogen bond network formed by K93, E123, and D253 with water molecules in CG also disappears. The CG change in the second and third cluster conformations in ChR2-cis decreases in order. Observing ECG suggests that the structure of the extracellular side of the central gate shows a complex H-bonding network among the residues D253, E123, K93, E97, E101, R120, and H249 in ChR2-trans. However, in the top three cluster conformations represented by ChR2-cis, the complexity and the number of hydrogen bonds are reduced compared with ChR2-trans. The number of hydrogen bonds in the first cluster conformation is much less than that of ChR2-trans. This indicates that the extracellular channel of the first cluster of ChR2-cis is more open.

The cluster analysis demonstrates that the ChR2-cis structure has significant changes at the three blockages of the ion channel compared with ChR2-trans. This is consistent with the expected trend of ion channel formation in the references [28,29]. Since the first cluster conformation is the largest proportion in the simulation trajectory and exhibits the most observed changes, the first cluster representative conformation of ChR2-cis is selected as the main conformation for the subsequent analysis after retinal isomerization.

**Figure 5:**
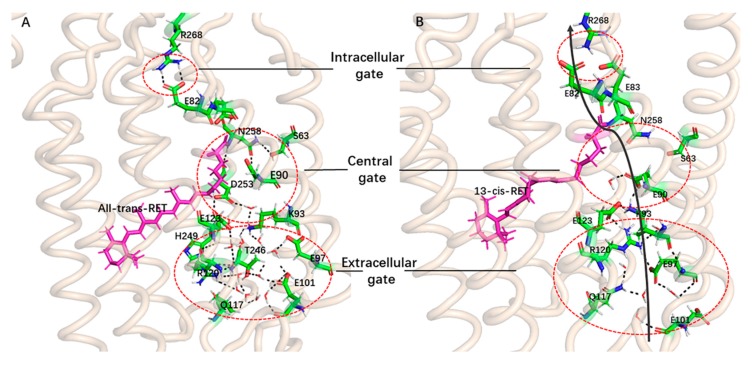
Structure comparison of ChR2-trans and ChR2-cis. (**A**) Structure of ChR2-trans in equilibrium trajectory. (**B**) The first cluster representative structure of ChR2-cis. In the figure, the black solid arrow represents the potential ion channel, the red dotted circle highlights the ICG, CG and the ECG in the pores of the protein channel. The hydrogen bonding between key residues is indicated by a black dotted line.

The stable ChR2-trans structure (Figure 5) is found to be the same as the reported crystal structure of ChR2 [35]. The CG in the central channel is also composed of residues S63, E90, D253, K93, and N258. The key amino acid E90 and N258 forms hydrogen bonding interaction and the D253, E123, and K93, are also connected through H-bond network mediated by water molecules. These block the central gate. Along from the cytomembrane to the intracellular side of channel, the ICG consists of residues E83, E82, and N258. There exists a double hydrogen bond between the residues R268 and E82, which blocks intracellular ion channel. In addition, as shown by the crystal structure of ChR2, the extracellular side of the channel is discontinuous. This is caused by the constrictions at the ECG through an extended H-bond network mediated by numerous water molecules. The ECG in the structure of ChR2-trans (Figure 5A) composed of Q117, R120, E97, E101, and H249, and contains numerous water molecules. The amino acids around the ECG are connected through the H-bond network mediated by these water molecules. This also causes the connection among the TM2, TM3, and TM7, and thus blocks the extracellular ion channels. It is also found in Figure 4 that the hydrogen bond network formed in the ECG is very complicated and extended to the CG. It is therefore concluded that ECG and CG are closely related.

**Figure 6:**
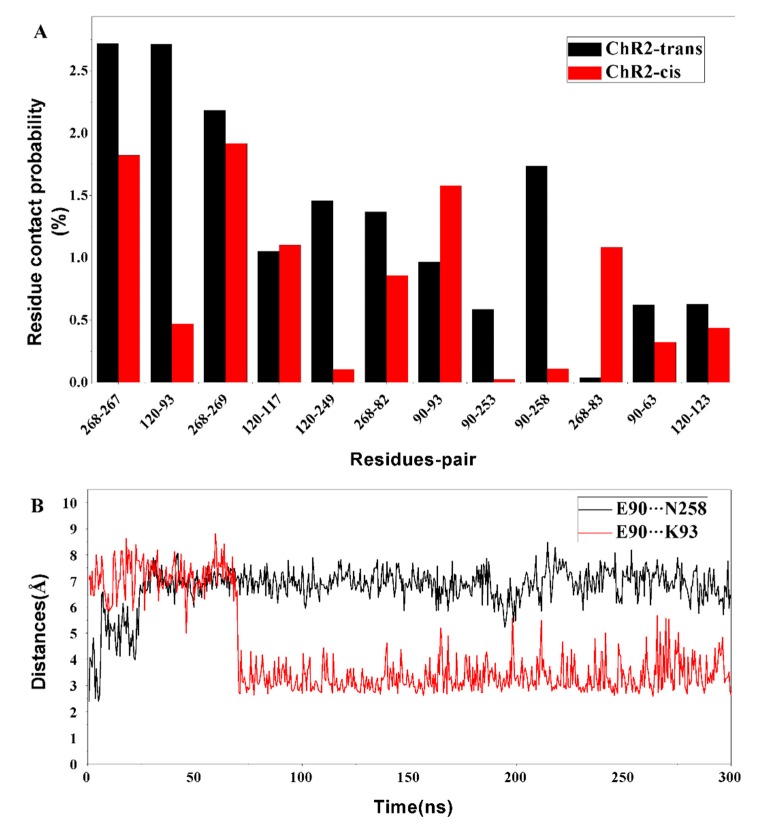
(**A**) The probability of contact among the key residues surrounding the channel in the equilibrium trajectories of ChR2-trans and ChR2-cis. (**B**) The hydrogen bond distance between E90 and N258 (black), as well as E90 and K93 (red) during the whole trajectory of ChR2-cis, the distance between E90 and N258 increases and the hydrogen bonding disappears; the distance between E90 and K93 is close to form a new hydrogen bond interaction.

The representative conformational analysis of ChR2-cis (Figure 5B) shows that retinal isomerization affects the adjacent N258 through the side chain of residues K257. This induces movement of the N258 side chain and results in weakening of the hydrogen bonds between E90 and N258. In addition, the outward flip of E90 further disrupts the H-bond interaction between N258 and E90. It is interesting that after the hydrogen bond between E90 and N258 breaks, the E90 more easily tilts outward and is then stabilized by a new connection with K93. To corroborate this observation, the probability of contact between the various residues around ion channel in the equilibrium trajectory of ChR2-trans and ChR2-cis was calculated (Figure 6A). Hydrogen bond distances between E90 and N258 as well as between E90 and K93 for the whole trajectory of ChR2-cis were also calculated and presented in Figure 6B.

It is seen that the hydrogen bond distances between E90 on TM2 and between N258 on TM7 increased remarkably for the first tens of ns, indicating the weakness of some H-bonding interaction. The contact probability of E90 and N258 in ChR2-cis is obviously lower than that in the closed-state ChR2-trans and is consistent with H-bonding interaction variation. After about 70 ns, the hydrogen bond distance between E90 and K93 on TM2 decreased dramatically and then remains stable with time. The contact probability between the E90 and K93 in ChR2-cis is larger than that in ChR2-trans over this range of time. These observations support the formation of the bridge between E90 and K93 after retinal isomerization. Original hydrogen bond interaction is destroyed in the CG and outward tilt of TM2 on the intracellular side enables water influx from the extracellular side into the channel. The hydrogen bond network in CG is also broken, opening a pore in the channel.

These changes in the central of ion channel support a previous assumption by Ardevol, Kuhne and Ruffert [28,29,40]: the downward orientation of E90 opens up a small pore and provide space for the influx of water molecules. This destroys the connection between TM2 and TM7, weakens the connection between E82 and R268 due to the displacement of TM2 at intracellular side, and consequentially leads to further opening of the channel. As seen in Figure 4 and Figure 5, the most important fact is that the key amino acid R268 of the intracellular gate is shifted away from the channel, directly destroying the hydrogen bonding interaction between R268 and E82 and prompting the formation of a more stable open state of ICG.

The crystal structure study of ChR2 [35] predicts that ions can pass through the channel only when all three gates (ECG, CG, ICG) remain open. The simultaneous opening of CG and ICG is discussed above. A brief description of the ECG opening based on the simulation results presented is outlined here. The residues K93, E97, E101, and M107 form a complex hydrogen bonding network with water molecules at the ECG and the network extends to D253 in CG. This leads to a close connection between ECG and CG. Since these amino acids are closely resided in TM2, the complex hydrogen bond network in ECG is inevitably destroyed by the relocation of TM2 due to the retinal isomerization. Eventually this opens the extracellular side of the channel, prompting the opening of ECG and CG simultaneously.

As analyzed above, the change of the crucial amino acid and the disruption of the hydrogen bond network after the retinal isomerization are critical for the opening of the channel. It can be seen in Figure 6A that the contact probability of some key residue pairs, such as E90 and N258, E82 and R268, in ChR2-cis are lower than those in ChR2-trans. This suggests that the changes in the conformation, displacement of the surrounding amino acids and the helix by retinal isomerization weaken the interaction of the crucial residue pairs of ChR2-cis.

**Figure 7:**
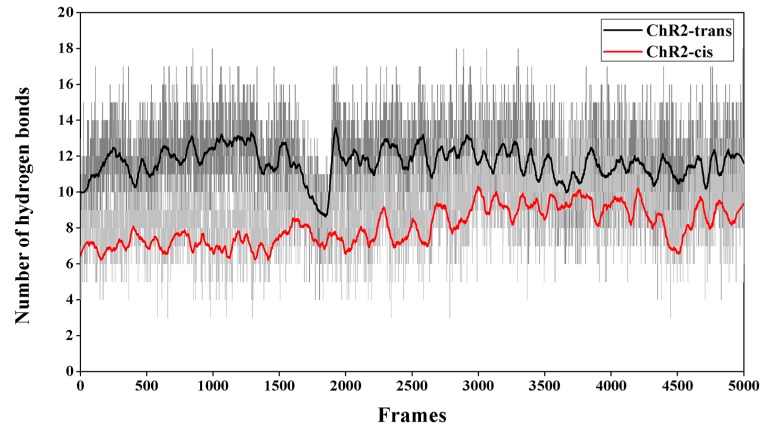
The number of hydrogen bonds between the residues and residues surrounding the channel (e.g., S63, E82, E83, E90, K93, E97, E101, Q117, R120, E123, T246, D253, N258, R268), as well as between residues and water molecules in the last 50 ns of equilibrium trajectory of ChR2-trans and ChR2-cis. It is apparent that ChR2-cis (red) has fewer hydrogen bonding interactions than ChR2-trans (black).

The number of hydrogen bonds between the residues and between residues and water molecules in the equilibrium trajectory of ChR2-trans and ChR2-cis was calculated and presented in Figure 7. It shows that the number of hydrogen bonds in the channel of ChR2-cis decreased significantly after retinal isomerization. This is also supported by the structural analysis in Figure 4 and Figure 5. It is therefore concluded that the retinal isomerization from all-trans to 13-cis is the main course of ion channel opening in ChR2-trans blocked by the interaction between residues and the formation of hydrogen bond network.

**Figure 8:**
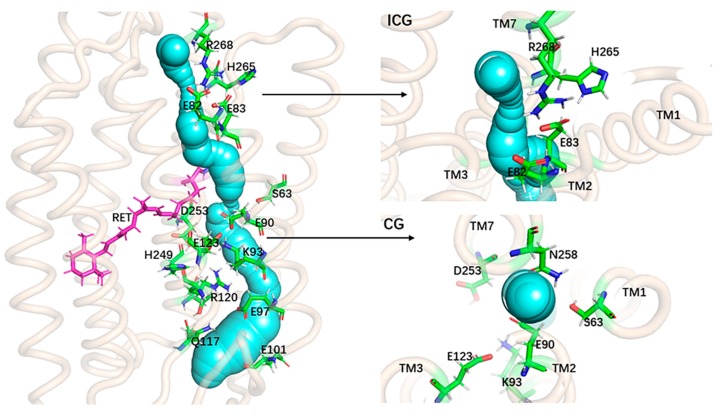
The static structure of the ion channel in the ChR2-cis. On the left is the overall view of the ion channel; the right side respectively is the side view of the channel at the ICG and the top view of the channel at CG.

To view the geometric structure of the opened-state channel, a static structure of the ion channel of ChR2-cis was calculated using the CAVER3.0 plug-in [42,43] and the results are summarized in Figure 8. The structure shows an obviously global shape of the ion channel. The opening and the stable cavity in the channel can be unambiguously viewed. The geometry of the ion channel and its surrounding amino acids can also be seen from the side and top views of ICG and CG respectively. Closer examination of Figure 8 suggests that the hydrogen bond destruction induced by the flipping and shifting of amino acids in the ChR2-cis provides space for the formation of ion channels in ChR2-cis.

To further understand the basic mechanism of ion permeation through the ion channel, the SMD and umbrella sampling approach were adopted to study the transmembrane process of the Na^+^ ion in the ChR2-cis.

**Figure 9:**
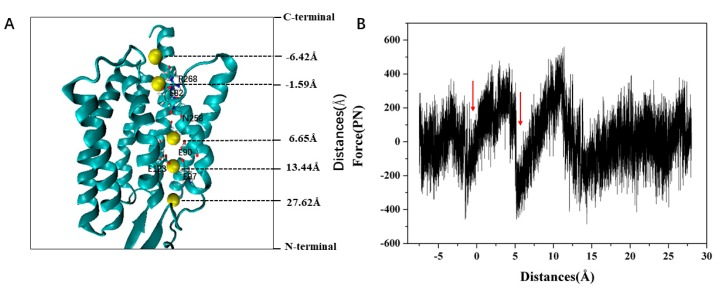
(**A**) The simulated trajectory of ion permeation through channels. Na^+^ is represented by the yellow sphere. (**B**) The force received varies with the reaction coordinates during ion pulling in the ChR2-cis system. The vertical arrow indicates the Z position of the force local minima.

Figure 9A presents a simulated trajectory of ion permeation through channels and Figure 9B presents the variation of the force received with the reaction coordinates during ion pulling in the ChR2-cis system. It can be seen that there are two notable local minima of force received by Na^+^ ion moving along the channel. The minimum of the force at z = −1.59 Å corresponds to the ICG with the electrostatic interaction between Na^+^ and E82, E83 in the channel (Figure 9A), which makes the force increase to about 400 pN as Na^+^ moves away from ICG. This result indicates that ICG plays an important role during the Na^+^ transmembrane process and can serve as a binding site of Na^+^. The second force local minimum is encountered at z = 5.65 Å within the CG region in the channel. Later, the force increases gradually and reaches a global maximum of about 500 pN at ~11 Å. The increased force can be attributed as follows: (1) Na^+^ can interact electrostatically with negative residues including E90, E123, and D253 at CG region, which makes Na^+^ hardly pass though CG; (2) the ion channel, as shown in the calculated static structure of the ChR2-cis (Figure 8), has a smaller bottleneck at the CG. The bottleneck makes Na^+^ suffer from more resistance during the transport process. Meanwhile, it can be seen from the Figure 9B that Na^+^ has no resistance on the extracellular side of the central gate.

The number of local minima and its coordinates found by the SMD simulation of the ChR2-cis system are in good agreement with the position of the protein binding site from the force change diagram of ions. When the Na^+^ ion moves through the ion channel, the stabilizing force is developed at the two binding sites, including ICG and CG. This is consistent with the expected change in the electrostatic interaction of Na^+^ with glutamic acid and aspartate side chains.

**Figure 10:**
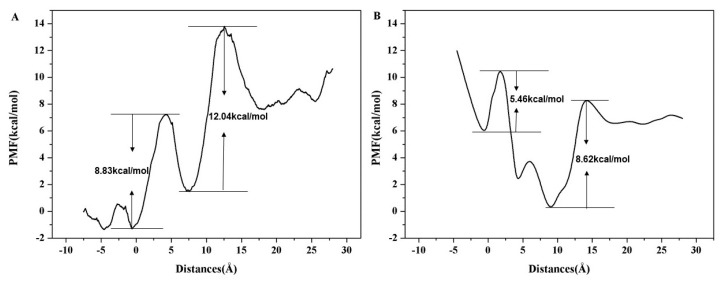
(**A**) PMF reconstructed used the Jarzynski equality and SMD trajectory for Na^+^ permeation across ions channel in ChR2-cis system. (**B**) PMF reconstructed use umbrella sampling for Na^+^ permeation across ions channel in ChR2-cis system.

To further examine the energy changes during the Na^+^ transportation along the ion channel, we calculated the potential of mean force (PMF) curve of Na^+^ based on the SMD trajectory and the Jarzynski equality was used to obtain equilibrium free energy. The PMF corresponds to the barrier a permeating ion has to overcome. It is crucial for the understanding of selectivity and conductivity of the ChR2. As shown in Figure 10A, there are two main energy potential wells in the penetration process of Na^+^. A potential well at the ICG (~–1 Å), PMF curve shows that 8.83 kcal/mol energy barrier needs to be overcome as Na^+^ passes through ICG. A deeper potential well was found at the CG (~6 Å) with a 12.04 kcal/mol energy. The calculated PMF are consistent with the binding sites of Na^+^ at the ICG and CG as revealed in Figure 9. This result indicates that the two energy wells partly hinder the penetration of Na^+^ into the ion channel.

We also performed umbrella sampling to validate the energy change obtained from the PMF curve of SMD simulation. As shown in Figure 10B, the PMF constructed by umbrella sampling is consistent with the PMF by SMD trajectory with two main energy potential wells at ICG and CG regions, respectively; the barrier is 5.46 kcal/mol at ICG and is 8.62 kcal/mol at CG. This result also indicates that the PMF obtained from SMD is reliable with the pulling velocity of 0.0001 Å·ps^−1^ as described in the Methods section. Obviously, the energy barrier at CG in both PMF curves is higher than that of ICG. Thus, the transport of Na^+^ in CG may serve as the rate-limiting step during the transmembrane transport process of Na^+^ in the ion channel of ChR2-cis. 

Potassium is a physiologically important ion and ChR2 is also known to conduct K^+^ [19] with lower selectivity than sodium. Therefore, we also checked the PMF curve of K^+^ for comparison. Since the main purpose of this work is to study the transmembrane transport of Na^+^, we only performed the SMD simulations to calculate the PMF of K^+^ transportation for a qualitative comparison with that of Na^+^. As with the Na^+^ system in SMD simulation, the pulling velocity for the K^+^ system was also set to 0.0001 Å·ps^−1^. As shown in Figure S6, compared with Na^+^, K^+^ needed to overcome a larger energy barrier to pass through the channel. Therefore, K^+^ is expected to have a lower conductivity than Na^+^, which is consistent with the experimental results [18,19,20,21]. We speculated that the conductivity was related to the ion radius. These simulation results provide valuable insights on the transient and dynamic interactions, and the precise location of the ion binding sites and the atomic details of the interactions between the ion and the channels and provide qualitative estimates of ion conductivity.

## 3. Materials and Methods

### 3.1. Simulation-System Preparation

The crystal structure of the ChR2 (ChR2-trans), as shown in Figure 2, was taken from the Protein Data Bank database (PDB ID: 6EID [35]). The coordinates of ChR2-cis were obtained as described by Takemoto [44]: the 13-cis retinal chromophore molecule was picked from the K-intermediate of bacteriorhodopsin (PDB ID: 1IXF [45]). The main chain atoms and the hexatomic ring of the 13-cis retinal molecule were superimposed on those of all trans retinal of ChR2, and then the all trans retinal was replaced with the 13-cis configuration in the ChR2-trans structure. Subsequently, a 15,000-step energy minimization was performed to generate the initial structure of the ion channel open state (ChR2-cis). The topology and force field parameters for 13-cis retinal were the same as those for all-trans retinal, which are also compatible with 13-cis retinal. The Membrane Builder in CHARMM-GUI software [46] was used to optimize the initial structures of ChR2-trans and ChR2-cis. Next, side chain was added for amino acid, implicit hydrogen atoms were added, protonated states were distributed, and the side chains of amino acids were optimized. Then the optimized structure was embedded in a pre-equilibrated 16:0/18:1c9-palmitoyloleyl phosphatidylcholine (POPC) lipid bilayer. The system was solvated with TIP3P water molecules and 0.15 mol/L of NaCl was used to model the experimental conditions. Finally, the system was simulated using periodic boundary conditions in a simulation box. The detailed information of each system is shown in Table 1.

### 3.2. Molecular Dynamics Simulations

The Classical Molecular Dynamics (CMD) and SMD were run in NAMD 2.12 [47] software with CHARMM36 force field. Long-range electrostatic interactions were treated with the Particle-Mesh-Ewald [48] approach with a grid spacing of less than 1 Å, and the cut-off value of short-range non-bond interaction is set to 12 Å. The bonds containing hydrogen atoms were frozen with SHAKE [49] constrain algorithm and a time step of 2 fs was used in the integration. The steepest descent method was used to minimize the energy of the system, and then the system was heated up in the NVT ensemble until the desired temperature of 310 K was reached. For this process, the pressure was kept at 1 bar using a Langevin Piston algorithm. Finally, all production runs were performed without any restraints under the constant temperature and pressure condition (NPT) at 310 K and 1 bar, where the temperature and pressure were maintained using the Langevin thermostat with a coupling coefficient of 1 ps^−1^ and the anisotropic Langevin piston barostat with a piston period of 50 fs and a decay of 25 fs. All membrane proteins, which undergo anisotropic structural changes between functional states, are likely to be affected by the lateral pressure profile. Therefore, we took the lateral pressure profiles for bilayers as the target characteristic and monitor the stability of the bilayer (see Figure S5 for detail). We used the integration time step of 2 fs, and the trajectories were saved at every 5 ps, with the last 50 ns used for the analysis.

### 3.3. Steered Molecular Dynamics

Although the development of computer technology has dramatically extended the time scale for dynamics simulation, the time scale of ion permeation through the ion channel is typically from milliseconds to seconds [27,28], which is definitely beyond the CMD capacity. In addition, the CMD tends to cause the sample to fall into a minimum energy well in the conformation space, making it difficult to achieve ion transmembrane conduction. For these limitations, SMD was adopted in this work [50,51,52,53,54] and an imaginary external force was artificially applied to Na^+^ in the simulation. This allows the ion to move along the channel. SMD gives the detailed information about the atomic coordinates during the stretching process. Important information about the process of ion transmembrane conduction is obtained by analyzing the structural and energy changes. This method is often used to study the transmembrane conduction of ions.

Generally, there are three ways to introduce external forces: simple harmonics potential (one-dimensional springs), surface tension, and torsion. In this work, a simple harmonic potential approach was used. SMD simulations were carried out as described with constant-velocity [55].

The initial structure in constant-velocity SMD is a representative configuration extracted from ChR2-cis cluster analysis. To enable it to move along the direction of the channel, Na^+^ was initially placed at the entrance of channel and was then pulled in the Z-direction (parallel to the channel axis) from the mouth to the center of the channel at constant velocities, but no restraint was imposed in the X and Y directions. In addition, SMD atoms can be pulled only with a hard spring at a constant velocity during the pull. The velocity was set to 0.0001 Å·ps^−1^ to ensure a reversible pulling and constant k was set to 4 kcal/mol·Å^−2^ to meet the requirements of hard spring. The detailed parameter adjustment is displayed in Figures S1–S4 in the supporting information. To avoid translation and rotation of proteins in SMD simulations, the Hamiltonian limit of 5 kcal/mol Å^−2^ was added to the amino acid residues surrounding the seven helices of the protein. Other parameters for SMD simulations are consistent with CMD. After that, the second order cumulant expansion formula of Jarzynski equation was used to construct PMF which relates the work of the non-equilibrium process to the free energy of the equilibrium process.

### 3.4. Determining the PMF with Umbrella Sampling

Since the shape of PMF constructed by SMD is slightly deformed during the pulling of the ion, and the accuracy of the curve is closely related to the pulling velocity, the system may not relax into an equilibrium state with an unreasonable velocity. Compared to SMD simulation, umbrella sampling [56,57] consists of running separate “windows” of the reaction coordinate simultaneously. From the sampled distribution of the system along the reaction coordinate, the change in free energy in each window can be calculated. The windows are then combined by methods like the weighted histogram analysis method or umbrella integration. Thus, the umbrella sampling simulation is capable of finding the equilibrium state in a set of umbrella sampling windows and obtaining relatively accurate PMF curve by reweighting constant biasing potential well along a reaction coordinate. To validate the reliability of PMF calculated from SMD simulation, we further performed additional umbrella sampling simulations for Na^+^ transport. 

The reaction coordinate used for the umbrella sampling simulations was the same reaction coordinate used for the SMD simulations, i.e., the z distance along the channel axis, and the reaction coordinate space is divided into windows every 1 Å. Thus, a total of 36 windows from −5 to 30 Å in the z direction were simulated independently. The SMD trajectory was used to generate the starting conformations for each independent window by extracting structures near the window centers. NAMD 2.13 was used to perform umbrella sampling simulation. A harmonic potential with force constant of 2.5 kcal/mol·Å is applied to maintain the respective distance in the z direction for each window, and the width of the Translocation colvar is 0.1 Å. As shown in Figure S7, these settings ensure a good overlap between neighboring windows. Finally, we used the Weighted Histogram Analysis Method (WHAM) [58] to construct the PMF based on these umbrella sampling simulation trajectories.

## 4. Conclusions

Classical molecular dynamics simulations on ChR2-trans and ChR2-cis were performed to study the formation mechanism of the cation channel of Channelrhodopsin-2. The results show that light excitation leads to all-trans to 13-cis isomerization of the ChR2 retinal chromophore. The conformation of key residues E90 and R268 are significantly changed because of the isomerization. This leads to the loss of the hydrogen bond interaction between E90 and N258 of CG, and between E82 and R268 of ICG. In addition, the variations in the number of hydrogen bonds between the residues and between the residues and water molecules in the equilibrium trajectory of ChR2-trans and ChR2-cis indicate that the H-bond network in the ChR2-trans structure is significantly destroyed because of the isomerization. It is therefore concluded that the retinal isomerization from all-trans to 13-cis causes the opening of ion channel in ChR2-trans blocked by the interaction between residues and the formation of hydrogen bond network.

The static structure of the ChR2-cis ion channel calculated demonstrates the stable cavities in the ChR2-cis. The force of Na^+^ passing through the ChR2-cis cation channel variation with the reaction coordinates showed that there are two binding sites at ICG and CG, and the potential mean force obtained by SMD and umbrella sampling stimulation indicated that energy potential wells at two binding sites partly hinder the penetration of Na^+^ into the ion channel.

## Figures and Tables

**Table 1:** Detailed information of the simulation system of ChR2-trans and ChR2-cis.

	Total atoms	Protein atoms	Phospholipid	Water	Ions	Periodic box (Å)
ChR2-trans	51,531	3957	17,286	30,232	56	73 × 73 × 104
ChR2-cis	52,838	3957	17,822	31,002	57	74 × 74 × 104

## Supplementary Materials

Supplementary Materials can be found at https://www.mdpi.com/1422-0067/20/15/3780/s1.

## Abbreviations

ChR2	Channelrhodopsin-2
ChR2-trans	all-trans-retinal configuration of ChR2
ChR2-cis	13-cis-retinal configuration of ChR2
CMD	Classical Molecular Dynamics
SMD	Steered Molecular Dynamics Simulations
