# Supplementary material for: Formation Mechanism of Ion Channel in Channelrhodopsin-2: Molecular Dynamics Simulation and Steering Molecular Dynamics Simulations
Source: Int J Mol Sci. 2019 Aug 2;20(15):3780. doi: 10.3390/ijms20153780 (PMC6695816; doi:10.3390/ijms20153780)
Supplement: Supplementary file 1 [file ijms-20-03780-s001.pdf]

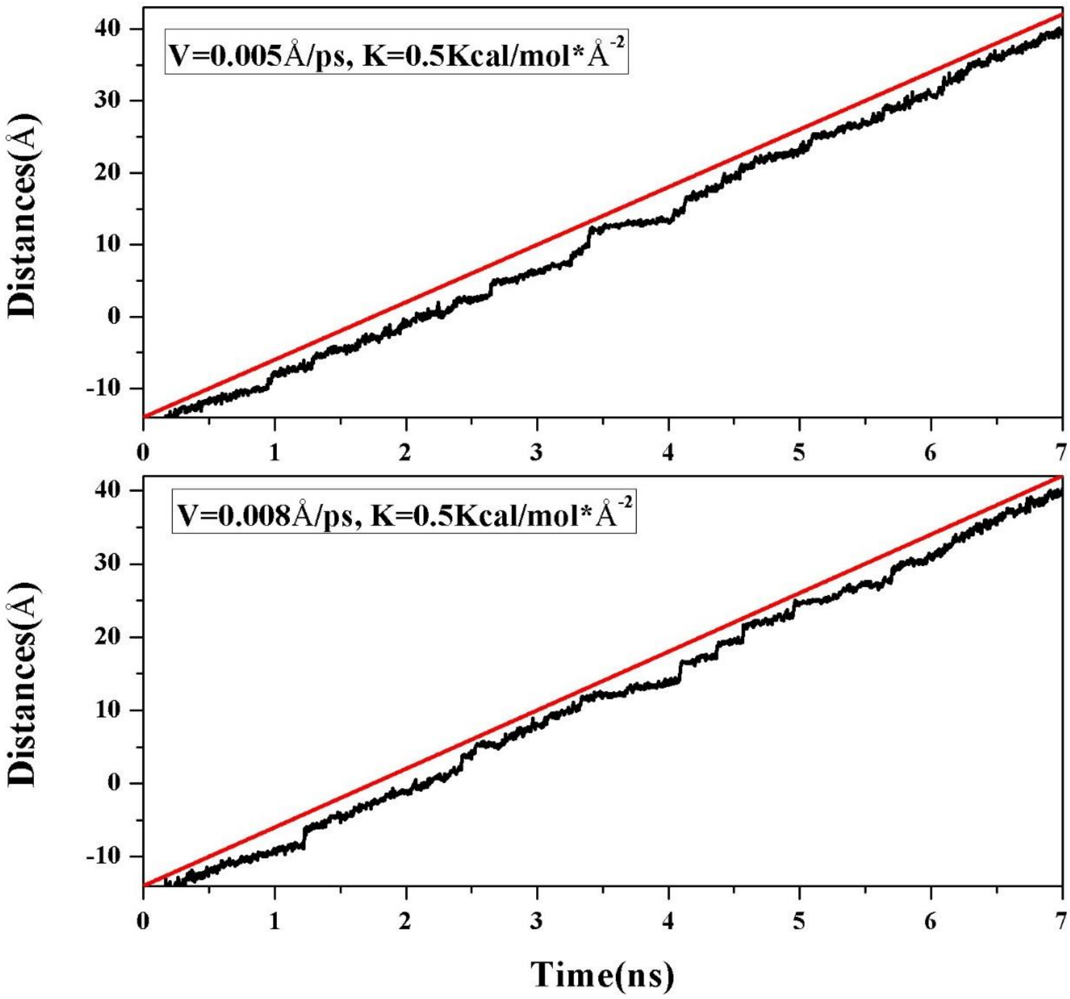

Figure S1. SMD parameter correction. For the ChR2-cis system, K=0.5 kcal/mol*$Å^{-2}$ remains unchanged, and V was set as 0.005 and 0.008Å/ps.

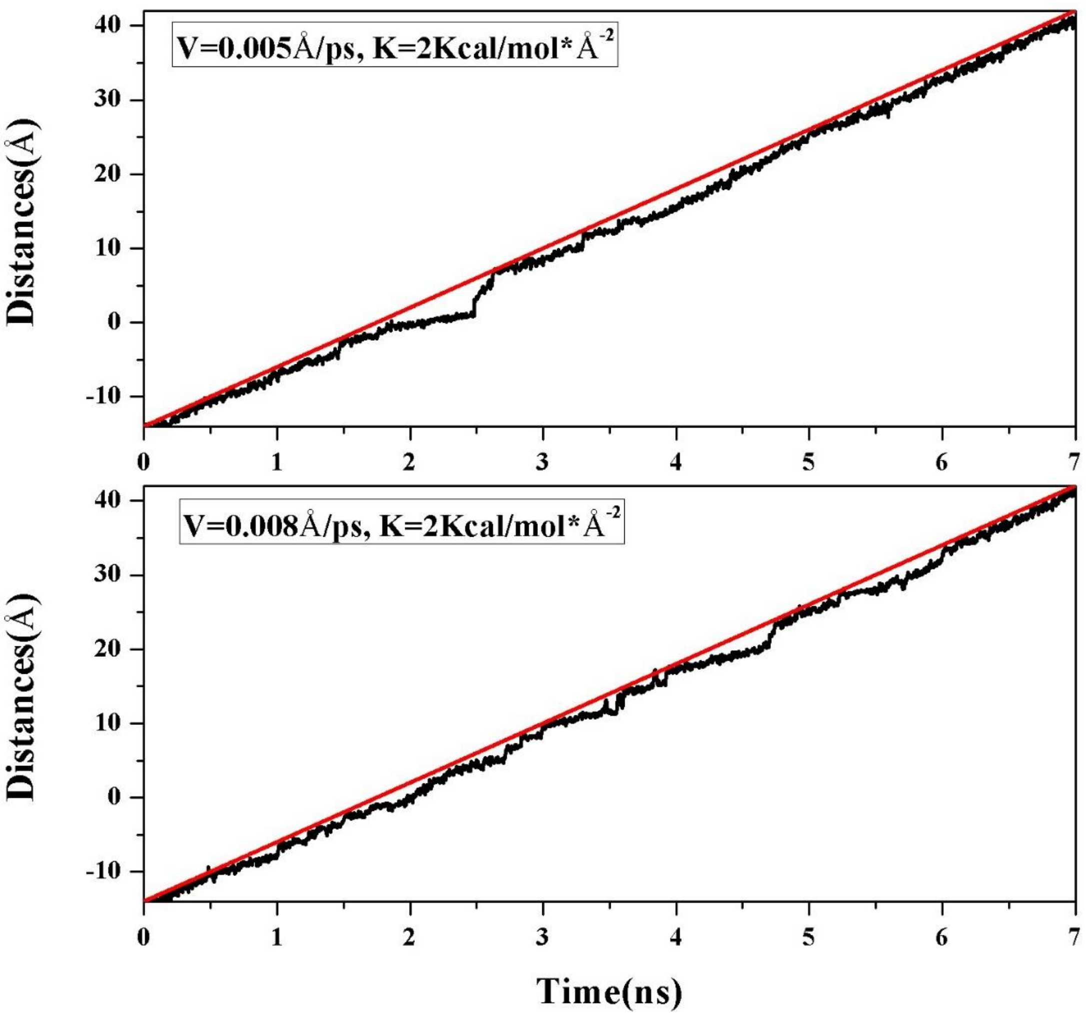

Figure S2. SMD parameter correction. For the ChR2-cis system, K=2 kcal/mol*Å^{-2} remains unchanged, and V was set as 0.005 and 0.008Å/ps.

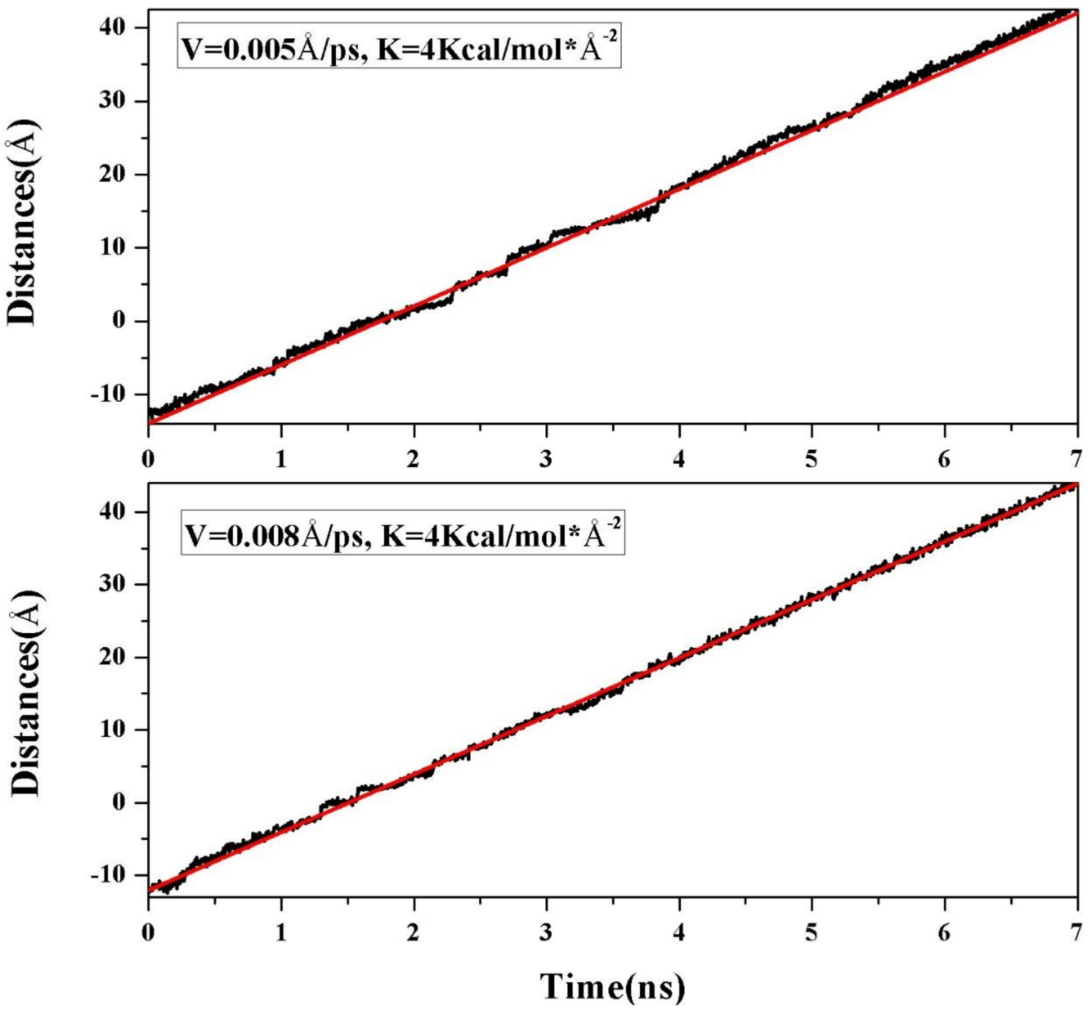

Figure S3. SMD parameter correction. For the ChR2-cis system, K=4 kcal/mol*Å^{-2} remains unchanged, and V was set as 0.005 and 0.008Å/ps.

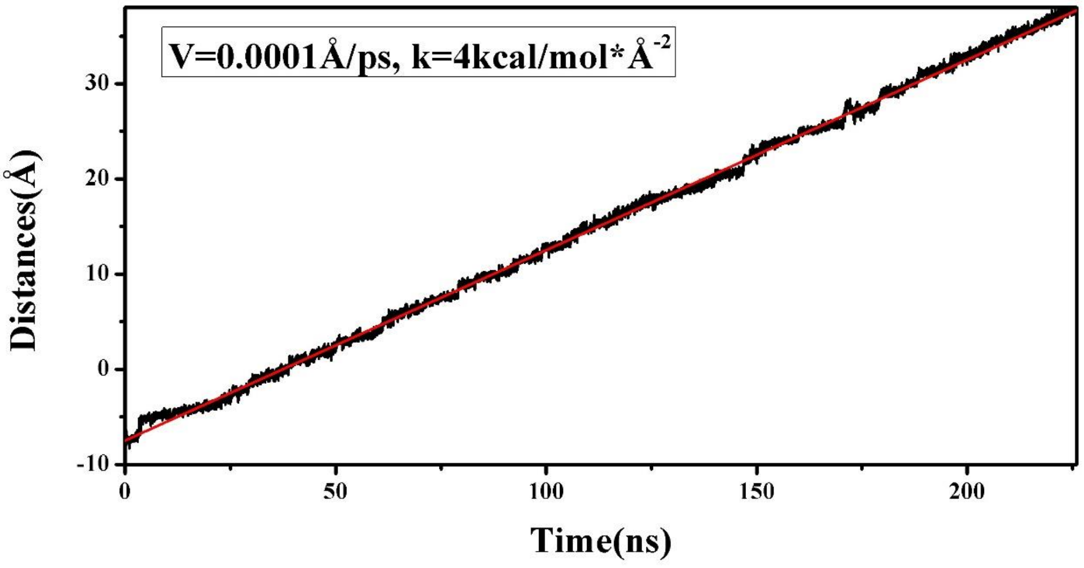

Figure S4. SMD parameter correction. For the ChR2-cis system, K=4 kcal/mol*Å^{-2} and V was set as 0.0001Å/ps.

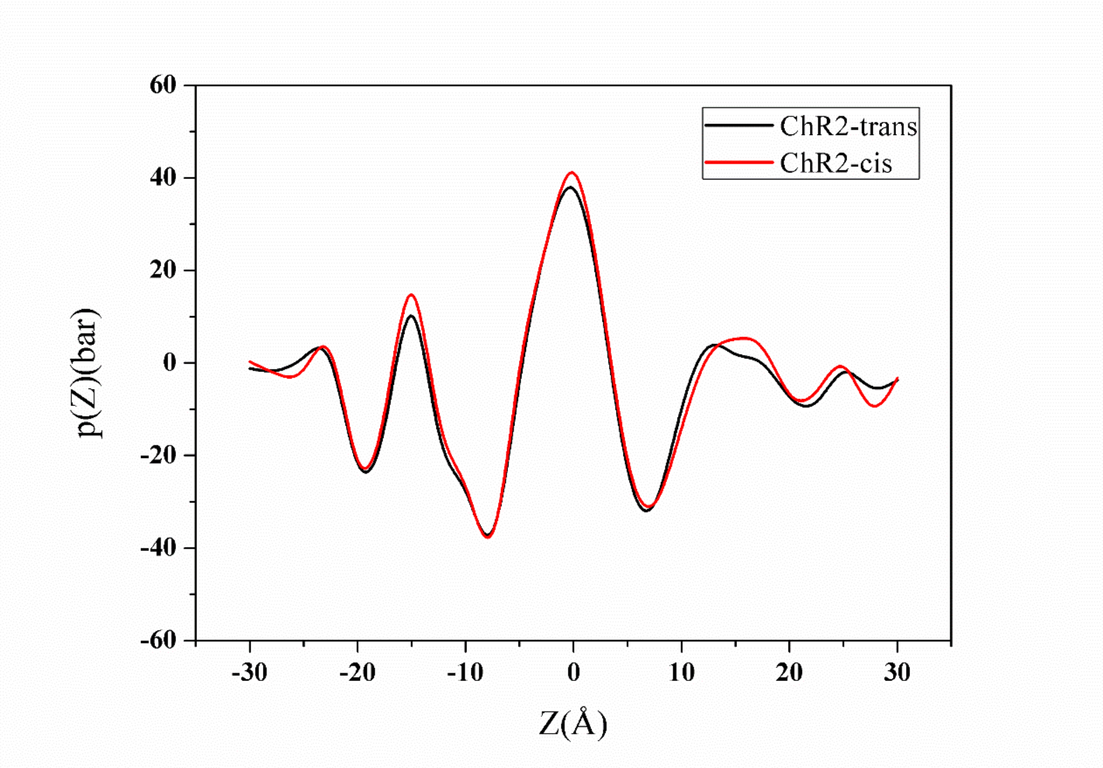

Figure S5. Total pressure profiles for POPC bilayers at the ChR2-trans (black) and ChR2-cis (red) systems. The z coordinate is defined normal to the bilayer with origin in the bilayer center of mass [1].

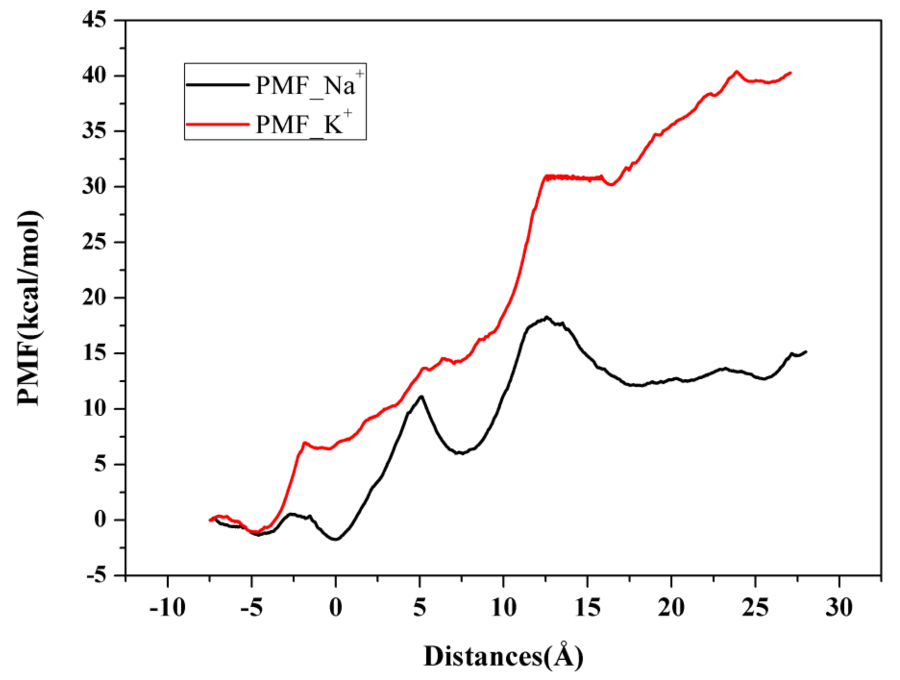

Figure S6. Potential of mean force (PMF) reconstructed used the Jarzynski equality and SMD trajectory for Na^+(black) and K^+(red) permeation across ions channel in ChR2-cis system.

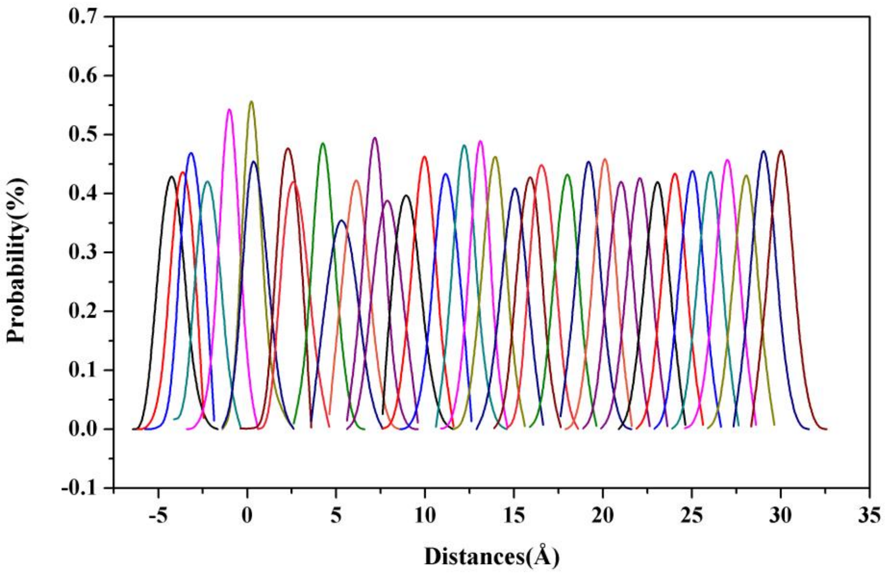

Figure S7. Histograms calculated from the trajectory data for the US simulations for every nanosecond.

References

1. Jacob, S.; Hansen, F. Y.; Peters, G. H. Methodological problems in pressure profile calculations for lipid bilayers. *J. Chem. Phys.* **2005,** 122, 124903.
